# mRNA/protein sequence complementarity and its determinants: The impact of affinity scales

**DOI:** 10.1371/journal.pcbi.1005648

**Published:** 2017-07-27

**Authors:** Lukas Bartonek, Bojan Zagrovic

**Affiliations:** Department of Structural and Computational Biology, Max F. Perutz Laboratories, University of Vienna, Campus Vienna Biocenter 5, Vienna, Austria; University of North Carolina at Chapel Hill, UNITED STATES

## Abstract

It has recently been demonstrated that the nucleobase-density profiles of mRNA coding sequences are related in a complementary manner to the nucleobase-affinity profiles of their cognate protein sequences. Based on this, it has been proposed that cognate mRNA/protein pairs may bind in a co-aligned manner, especially if unstructured. Here, we study the dependence of mRNA/protein sequence complementarity on the properties of the nucleobase/amino-acid affinity scales used. Specifically, we sample the space of randomly generated scales by employing a Monte Carlo strategy with a fitness function that depends directly on the level of complementarity. For model organisms representing all three domains of life, we show that even short searches reproducibly converge upon highly optimized scales, implying that the topology of the underlying fitness landscape is decidedly funnel-like. Furthermore, the optimized scales, generated without any consideration of the physicochemical attributes of nucleobases or amino acids, resemble closely the nucleobase/amino-acid binding affinity scales obtained from experimental structures of RNA-protein complexes. This provides support for the claim that mRNA/protein sequence complementarity may indeed be related to binding between the two. Finally, we characterize suboptimal scales and show that intermediate-to-high complementarity can be reached by substantially diverse scales, but with select amino acids contributing disproportionally. Our results expose the dependence of cognate mRNA/protein sequence complementarity on the properties of the underlying nucleobase/amino-acid affinity scales and provide quantitative constraints that any physical scales need to satisfy for the complementarity to hold.

## Author summary

Messenger RNAs and proteins, two essential types of biopolymers, have recently been shown to exhibit closely related, complementary physicochemical properties. Specifically, density profiles of certain groups in messenger RNA sequences directly match the affinity profiles for precisely those groups in protein sequences they encode. Based on this, it has been suggested that these molecules may interact with each other specifically and in a co-aligned fashion, especially when unstructured. Here, we explore different amino-acid scales used in the above analysis to assess which of their properties dictate the observed matching. Specifically, we define the constraints that need to be satisfied by physical scales for the complementarity to hold and show that the previously derived nucleobase/amino-acid affinity scales indeed satisfy these constraints. As a whole, our work provides a quantitative foundation for understanding the putative messenger RNA/protein complementarity with implications in different areas of RNA/protein biology including transcription, translation, splicing and viral assembly.

## Introduction

**Fig 1:**
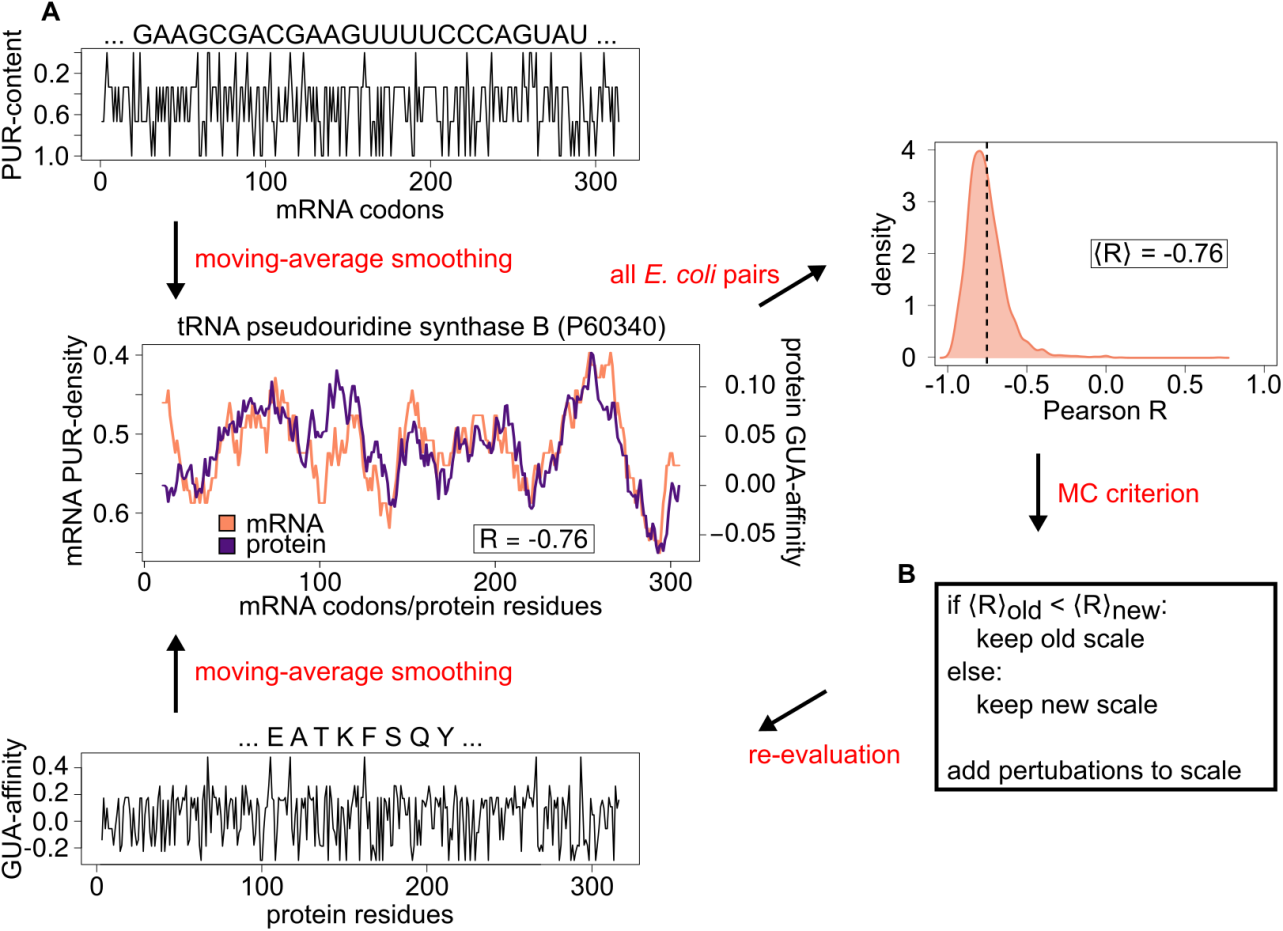
Cognate mRNA/protein sequence profile comparison and scale optimization. A) *top and bottom*: mRNA nucleobase-density profiles are obtained by window-averaged smoothing of their nucleobase composition strings, while nucleobase-affinity profiles of their cognate proteins are obtained by weighting their sequences with the aid of nucleobase-affinity scales and subsequent window-averaged smoothing; *center*: comparison between an mRNA PUR-density profile and the cognate protein’s GUA-affinity profile for a typical, representative mRNA/protein pair in *E*. *coli*. Matching between the two profiles (Pearson R = -0.76) corresponds to the mean Pearson correlation among all cognate mRNA/protein pairs in *E*. *coli*; *right*: distribution of Pearson Rs for all E. coli cognate mRNA/protein pairs. Note that the scale used for these profiles is the knowledge-based scale derived from the structures of RNA-protein complexes. In our simulation, we have explored the full space of such scales as shown in the text; B) pseudo code of the Monte Carlo strategy employed in the simulation.

The relationship between mRNAs and the proteins they encode is one of the key defining characteristics of life at the molecular level [1–3]. While this relationship has primarily been studied in the context of biological information transfer, less attention has been paid to a potential link between the physicochemical properties of the two biopolymers. Recently, however, we have reported an unexpectedly strong connection between the nucleobase-density profiles of mRNA coding sequences and the nucleobase-affinity profiles of their cognate proteins [4–6]. For example, purine (PUR) density profiles of *E*. *coli* mRNA coding sequences match their cognate protein’s guanine (GUA) affinity profiles with an average Pearson correlation coefficient of -0.76 (note the negative values for R indicate matching as a result of the standard definition of binding affinities) [4,5]. As illustrated in Fig 1A, the protein GUA-affinity profiles in this analysis were calculated by weighting their sequences with the GUA-affinity values for individual amino acids, which in turn were derived from known 3D structures of RNA/protein complexes by using a knowledge-based formalism [4]. In addition, we have also studied other affinity scales derived by diverse experimental and theoretical approaches: 1) a chromatographically determined scale of amino-acid affinity for pyrimidine (PYR) mimetics pyridines [7], 2) a computationally derived variant of the same scale [8], 3) absolute binding free energy scales between nucleobases and amino-acid sidechain analogs in different solvents [9], and 4) affinity scales obtained from simulated partitioning experiments using realistic RNA nucleobases [10,11]. The consensus of these studies has been that the mRNA regions rich in a particular nucleobase or a type of nucleobases (PUR or PYR) tend to encode the protein regions with a pronounced affinity for precisely those or similar bases. This novel finding is well illustrated by the mRNA PUR-density profile and its cognate protein’s GUA-affinity profile of a typical, representative mRNA/protein pair in *E*. *coli* whose Pearson correlation coefficient (-0.76) equals that of the mean of the entire *E*. *coli* distribution (Fig 1A). Importantly, the only exception to the above rule was seen in the case of adenine (ADE). Namely, protein regions with a high affinity for the purine base ADE tend to be encoded by mRNA regions rich in PYR bases [5,6].

Although robust and of potentially far-reaching implications, the above findings still lack a definitive explanation. We have suggested two possibilities: one, concerning the reasons for the observed complementarity, and another one, concerning its implications. First, the above observations are consistent with a possibility that genetic encoding may have arisen from binding, as postulated by the stereochemical hypothesis of the origin of the genetic code [2,12–13]. This hypothesis dates back to 1960s, but we believe our results provide the strongest-yet evidence for it. Importantly, however, our results shift the focus towards analyzing the binding in the context of longer biopolymers and not just isolated codons and amino acids [4–6,14]. Second, the compositional mirroring between mRNAs and their cognate proteins at the level of primary sequences supports the possibility of complementary, co-aligned binding between the two even in modern systems, especially if they are unstructured [4–6,14]. On the side of proteins, this pertains to both intrinsically disordered proteins as well as the unfolded states of otherwise folded proteins, such as during translation or upon thermal/chemical stress. While we do not exclude the possibility of interactions in the structured states of either partner [15], this also more likely involves mRNA stretches that are not base-paired. Overall, the potential implications of this interpretation concern different facets of RNA/protein biology including translation control, structure of ribonucleoprotein particles, the behavior of non-membrane-bound cellular compartments, viral capsid assembly and others [5]. More than two decades ago, Kyrpides and Ouzounis proposed that cognate mRNA/protein interactions may be an ancient mechanism for auto-regulation of mRNA stability [16]. Our findings now provide mechanistic details behind such a possibility. Here it should also be mentioned that the opposite behavior of ADE suggests that there may have been at least two major phases in the code’s development, the more recent of which introduced ADE to modulate in a negative manner the complementarity engendered by other bases [5].

The statistical significance of the cognate mRNA/protein sequence complementarity has already been probed by different tests involving randomizations of the genetic code table, shuffling of both the affinity scales and the primary mRNA/protein sequences and analyzing the behavior of other physically realistic amino-acid property scales in the same context [4,6,14]. However, these approaches, although valid and necessary, have thus far not included a systematic exploration of the whole space of nucleobase/amino-acid affinity scales. On the other hand, several important questions can only be answered with an in-depth knowledge of the properties of the space of affinity scales and their influence on the observed complementarity. A key open problem in this regard concerns the uniqueness of the affinity scales that yield a high degree of cognate sequence-profile matching. Is there an optimal scale that results in maximized matching between mRNA nucleobase-density profiles and their cognate protein sequence profiles or are there multiple scales that produce the same or similar levels of matching over complete proteomes? How many different scales are there that show intermediate-to-high levels of matching? Finally, are there specific amino acids whose affinities for different nucleobases dominate the matching? It is easily imaginable that there are amino acids that exhibit similar affinities to different nucleobases, while the specificity i.e. the sequence complementarity, is dictated by a select few. To address these questions, one needs to consider not only the already published, physically realistic scales, but also those that do not produce strongly matching profiles. In other words, one would like to sample the space of randomly-generated scales using a fitness function that is related to the degree of cognate mRNA/protein sequence complementarity engendered by those scales.

Here, we present a Monte Carlo (MC) search method that satisfies the above criteria and explores the space of nucleobase/amino-acid affinity scales, while at the same time assessing their impact on the degree of cognate complementarity (Fig 1A and 1B). More specifically, our MC searches start from a uniform scale with identical weights for all 20 amino acids and evolve through a succession of random perturbations, which are accepted or rejected according to a fitness function. The latter, in turn, is related to the degree of complementarity as captured by the proteome-average Pearson correlation coefficient <R> between the cognate sequence profiles (see Materials and Methods for details). In other words, we search for amino-acid scales (i.e. 20-element linear arrays of amino-acid weights), which result in a given level of proteome-wide average matching between mRNA nucleobase-density profiles and the cognate protein profiles generated by weighting their sequences using these scales. Notably, our procedure is completely computational and does not impose any physicochemical constraints on the sampled scales. As a consequence, it provides an unbiased, detailed characterization of the space of amino-acid scales and their effect on the cognate mRNA/protein sequence complementarity. Finally, there are ongoing efforts in our and other laboratories to test the hypothesis that compositional sequence complementarity between mRNAs and their cognate proteins implies binding between them. The primary aim of the present work, however, is to assess the impact of affinity scales on such sequence complementarity, which remains a fact even in the absence of experimental verification of the hypothesis that it implies binding [4–6,9,11].

## Materials and methods

### Data sets

Complete annotated proteomes of *Escherichia coli*, *Methanocaldococcus jannaschii* and *Saccharomyces cerevisiae* along with the corresponding mRNA coding sequences were analyzed. The protein sequence data was extracted from the UniProtKB database with the maximal-protein-evidence-level set to 4, including only reviewed Swiss-Prot entries for the analysis [17–19]. Coding sequences for each protein were extracted using the ‘Cross-references’ section of each entry in the UniProtKB. Of all the entries, the first one satisfying the length criterion of mRNA length = 3 x protein length + 3 was selected and the sequence downloaded from the European Nucleotide Archive Database. All sequences containing non-canonical amino acids or nucleobases were disregarded in the analysis. The complete sets of mRNA/protein data used in this study are included in S1 Dataset. Note that in the present study our analysis is reserved for primary sequences of complete mRNA coding sequences and their cognate proteins only, without consideration of higher-order structural organization. This not only enables a direct 1-to-1 mapping between mRNA and protein sequences, but also allows for a full exploration of the complementarity hypothesis in the unstructured context. Analysis of the influence of structure on the side of protein has been published elsewhere [15], while an analogous analysis on the side mRNAs will be the topic of our future work.

### Correlation calculation

The mRNA nucleobase-density profiles and the corresponding protein nucleobase-affinity profiles were compared by calculating the linear Pearson correlation coefficients R between them. Prior to calculation, the sequences were smoothed via a window-averaging procedure with a 63-nucleotide window for mRNAs and a 21-residue window for proteins as used before [4–6,20]. Importantly, sequence profile comparison is largely insensitive to the size of the averaging window, as shown previously [4]. Furthermore, the scale values obtained during the simulation may result in any arbitrary value including negative numbers. Once the simulation is finished, the resulting scales are rescaled so that the values are in the range of [0, 1] i.e. the lowest value is set to 0, the highest to 1 and the rest are rescaled accordingly. This is done in order to distinguish between truly different scales and the rescaled versions of the same scale. Importantly, this procedure has no impact on our analysis since both window-averaging and calculation of Pearson correlation coefficients are invariant with respect to linear rescaling.

### Monte Carlo simulations

The simulations were carried out using a combination of a C++ program for calculating the proteome-average correlation coefficients between mRNA and protein sequence profiles [6] and a Python script for implementing the Monte Carlo (MC) search. At each step in a given MC simulation, anywhere between 1 and 4 randomly chosen scale values were perturbed by a randomly chosen offset. A simulated annealing procedure was implemented in order to vary the size of the offsets from a randomly chosen value between [-0.1, 0.1] in the beginning of the simulation to a value between [-0.01, 0.01] in the end, with a linear ramp between the two. A given MC move i.e. a given scale, is accepted according to a zero-temperature, downhill Metropolis criterion: if a new scale results in a lower average Pearson R across the proteome (<R>) as compared to the scale it was derived from, it is accepted, and it is rejected otherwise. Affinity scales were generated individually for each of the four RNA nucleobase (uracil—URA, cytosine—CYT, adenine—ADE and guanine—GUA) as well as PUR (preference for both ADE and GUA). Given that the mRNA PYR fraction in a given stretch is directly related to the PUR fraction (%PYR = 1—%PUR), the PYR scales are by definition the inverses of the PUR scales and were for this reason not explicitly included in our analysis. Finally, the number of steps and the speed of simulated annealing both influence the final result. The number of steps has been chosen to be at least 3 times the number of moves necessary to reach a stable minimum.

### Selection of scales for landscape generation

The MC approach does not produce interaction scales for a given level of matching but the other way around–only once a scale has been created, its level of matching is calculated. In order to generate the full landscape, we chose those scales that showed the closest value of matching to the target value of the reported Pearson R. In the construction of the landscape, we include individual scales that are within +/-0.01 in Pearson R from each reported value of <R>. Considering the very fast evolution of interaction scales, for some low values of <R> we did not obtain a full set of 1000 scales that would match this criterion, which were therefore not included.

### Analysis and software used

Data analysis was performed using the R statistical programming language. Calculations were performed using custom software written in C++ and Python. Plotting and data visualization was performed in R and Python, while figures were generated in Gimp and Inkscape.

### Statistical significance tests

The MC-optimized scales were compared with the corresponding physically realistic knowledge-based scale by calculating the Pearson correlation coefficient R between them. The significance of the obtained correlation coefficients was ascertained by a randomization procedure whereby the reported p-values correspond to the fraction of a set of 10^6^ scales with randomly chosen values exhibiting a more negative Pearson R than the MC-generated scales (a one-tailed significance test). Two-tailed p-values were calculated by multiplying the initial p-value by 2 if below 0.5, or as 1—p-value otherwise. Combined p-values were calculated according to Fisher’s method based on two-tailed p-values [21,22]. The p-values were calculated from their respective Χ² distributions utilizing Microsoft Excel’s function CHIDIST().

## Results

Optimized scales, which lead to a close matching between mRNA nucleobase-density profiles and sequence-weighted cognate protein profiles, could be identified in an extremely low number of MC moves. For example, the number of MC moves required to reach a proteome-wide average matching of mRNA PUR-density profiles with <R> ≤ -0.86 in *E*. *coli* is only 322 ± 66.5 (standard deviation) as evaluated over 1000 independent MC simulations initiated with the system time as a random seed for each run (S1 Fig). Importantly, for all the combinations tested, the optimal scales appear to be extremely well defined and emerge robustly at the end of all independent MC simulations (scales with average weights are given in S1 Table). Also, the equivalent optimized scales for the three organisms studied are highly similar to each other with pairwise Pearson Rs in all cases exceeding 0.86 (S1 Table). The minor deviation between the scales of different organisms can be explained by the codon usage bias as well as different amino-acid composition of the respective organisms. For example, the difference between CYT_*E*. *Coli*_ and CYT_*M*. *Jannaschii*_ can be traced back to the different choice of codons for arginine.

**Fig 2:**
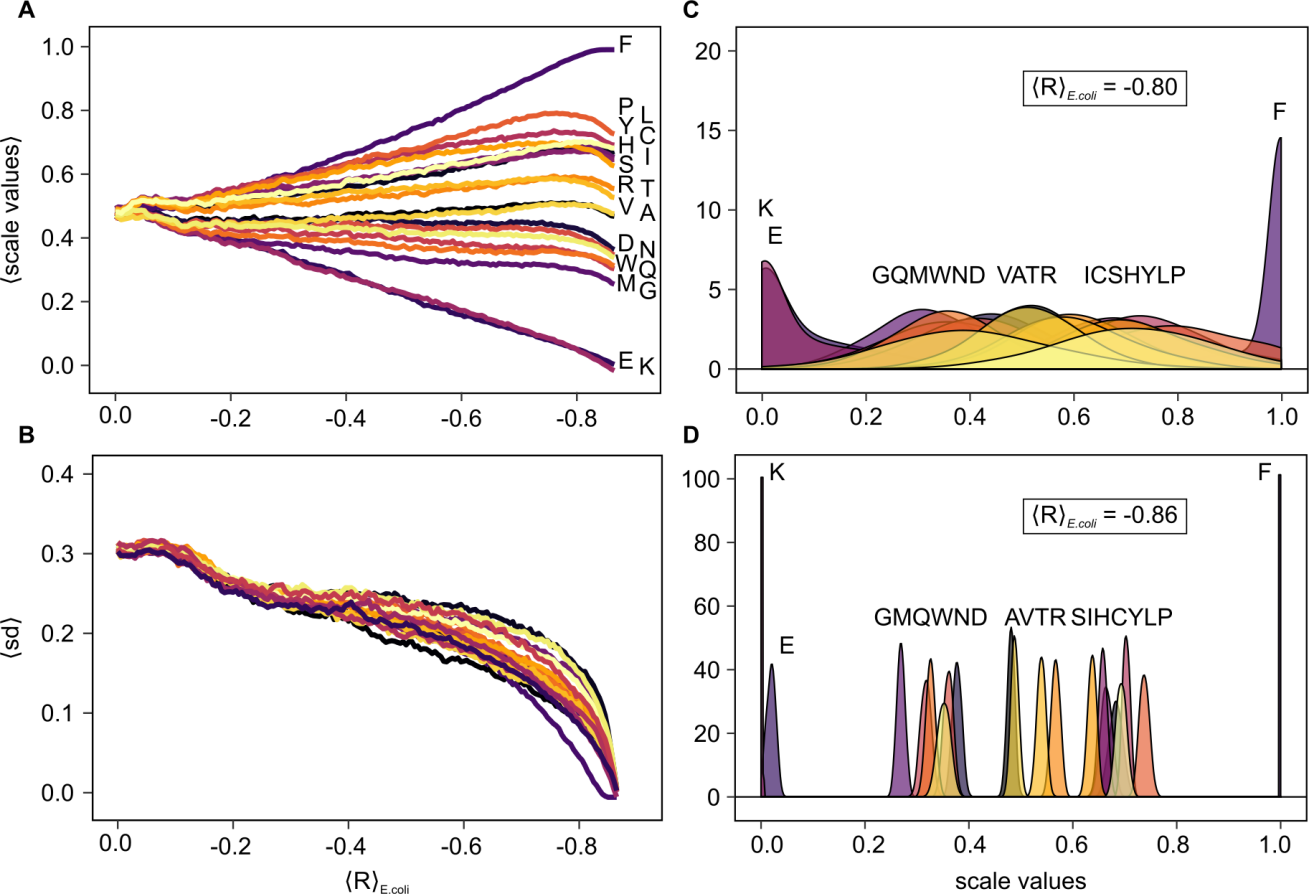
Dependence of profile matching on scale properties. The average level of cognate matching between mRNA PUR-density profiles and cognate protein PUR-affinity profiles in *E*. *coli* in relation to A) the average and B) the standard deviation of the rescaled PUR-affinity values for each amino acid yielding the given level of matching. The color code in B is the same as in A; C) distribution of PUR-affinity scale values at a matching level of <R> = -0.80; D) distributions of values of the best-matching PUR-affinity scales generated in 1000 different MC simulations (<R> = -0.86). Panels A,B and C,D share the same x-axis.

In Fig 2A and 2B, we trace the evolution of the average and the standard deviation of the normalized values of individual amino-acid weights corresponding to a scale that was optimized for matching the mRNA PUR-density profiles in *E*. *coli*. The mean weights corresponding to the majority of amino acids, as obtained from 1000 independent repetitions, exhibit well-defined ranks starting already with low levels of matching and attain their definitive ranks already at approximately <R> = -0.5 (Fig 2A). In this particular case, the extreme weights correspond reproducibly to Phe on the high side and Glu and Lys on the low side. The reproducibility of optimal scales is attested by the extremely low standard deviations of amino-acid weights at the extremely high values of <R> (Fig 2B). Importantly, although a sharp drop in standard deviation towards high levels of matching is observed, a clear trend for the specific values is achieved only very late in the simulations i.e. for the most extreme values of <R> only. Expectedly, the highest diversity of scale values is obtained for <R> ~ 0, but a substantial variability is retained even for intermediate-to-high values of matching: e.g. for <R> between -0.4 and -0.6, the standard deviations remain close to 0.25 in normalized units (Fig 2B). For comparison, a uniform random distribution between 0 and 1 results in a standard deviation of 1/√12 ~ 0.29.

The sequences of mRNAs and their cognate proteins are, of course, linked by the universal genetic code. Therefore, suitable amino-acid scales for weighting protein sequences to match their cognate mRNA’s density profiles correspond, for a particular nucleobase, to the relative fractions of that nucleobase in the respective codons, weighted by the codon usage bias. For example, the scale derived in such a way results in an average matching of <R> = -0.86 for the mRNA PUR-density profiles in *E*. *coli* and the cognate protein sequences weighted by the average, usage-bias-weighted PUR content of the respective codons. Equivalent levels of matching were obtained for all nucleobases and organisms (Table 1). Interestingly, the scale composed of the average MC-optimized weights derived from *E*. *coli* mRNA PUR-density profiles correlates with the scale derived from codon PUR fractions with a Pearson R = 0.997 (S1 Table). Similar results were obtained for all other nucleobases and in all other organisms, albeit with slightly lower levels of correlation in some cases (S1 Table).

**Table 1:** Minimum <R> values achieved for each organism for the MC-optimized scales.

	ADE	CYT	GUA	URA	PUR
***E*. *coli***	-0.89	-0.75	-0.84	-0.80	-0.86
***M*. *jannaschii***	-0.90	-0.85	-0.89	-0.87	-0.92
***S*. *cervisiae***	-0.87	-0.78	-0.86	-0.84	-0.89

The main advantage of the MC approach, besides its efficiency, is that it also provides thorough sampling of suboptimal scales. This has allowed us to explore the development of scale properties in relation to the degree of mRNA/protein sequence complementarity. When looking at the distribution of specific values for a given level of <R> over the whole set of mRNAs and proteins, strong differences between the final optimized values and the intermediate values are identified. In Fig 2C and 2D, we show the complete distributions of weights for different amino acids at two different levels of average matching in *E*. *coli* (<R> = -0.80 and <R> = -0.86) for the PUR-density scales. Here, from each independent simulation, the scale resulting in <R> closest to the target value was selected. Note that the <R> values of all selected scales round to the reported target value at the second decimal place. As can be seen, only the most extremely optimized scales i.e. those with <R> = -0.86, exhibit well-defined weights for the majority of amino acids. For example, although an average Pearson <R> of -0.80 can be considered a high level of matching, most scale weights are still broadly distributed at that level (Fig 2C). Importantly, different amino acids exhibit distributions of different widths at a given value of <R>, with some converging to tighter distributions earlier than others. For example, the weights for Phe, Lys and Glu attain their final values early on and exhibit standard deviations that are lower than for any other amino acids at most values of <R>. In general, the distinct behavior of the optimized weights corresponding to individual amino acids is also seen for other scales and in other organisms (S2 Fig).

**Fig 3:**
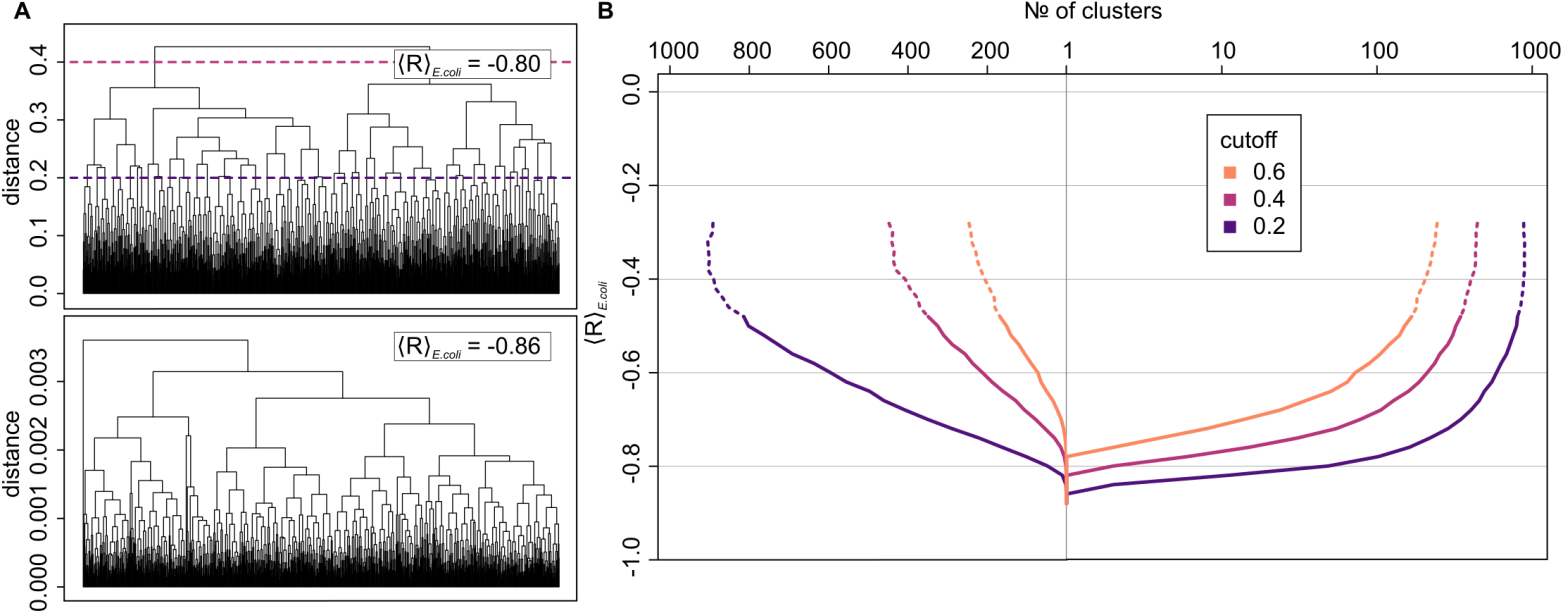
Landscape of affinity scales. A) two dendrograms exemplifying the process of clustering of scales for landscape generation (top, <R> = -0.80; bottom, best level of matching with <R> = -0.86). The height of the barrier between two clusters (left axis) is given as distance = 1—R between two scales. The presented results are obtained for mRNA PUR-density profiles and cognate protein PUR-affinity profiles in *E*. *coli*. The dotted lines capture the cutoffs of 0.2 and 0.4 used in Fig 3B; B) the number of clusters of scales at a given average level of matching as obtained by Pearson R-based clustering (left: linear scale; right: logarithmic scale) at different values of the upper cutoff for the distance (1—R) of two scales in one cluster.

How many distinctly different scales are able to perform similarly well when it comes to the sequence profile matching of mRNAs with their cognate proteins? To separate scales at a given level of matching into several subsets, we have applied a hierarchical clustering algorithm, which has allowed us to build dendrograms for each level of <R>. As a natural distance measure between scales in this clustering approach, we have used 1—R, with lower numbers signifying higher similarity between two given scales and *vice versa*. In Fig 3A, we show two such dendrograms capturing the diversity of scales obtained by matching mRNA PUR-density profiles in *E. coli* at <R> = -0.8 and <R> = -0.86. To build a landscape of affinity scales, we have cut these dendrograms at specified distance cutoffs and have reported the number of clusters at a given cutoff as exemplified in Fig 3A for *E*. *coli*. The landscape is presented in Fig 3B with the values given representing the upper cutoff for the distance (1—R) of two scales in one cluster. From this landscape, it is clear that only at the highest levels of correlation between mRNA nucleobase profiles and nucleobase-affinity profiles of their cognate proteins do the affinity scales show a very similar structure. At lower levels, a very diverse set of scales is able to perform comparably well i.e. the lower values of matching can be attributed to a wider class of interaction scales.

**Fig 4:**
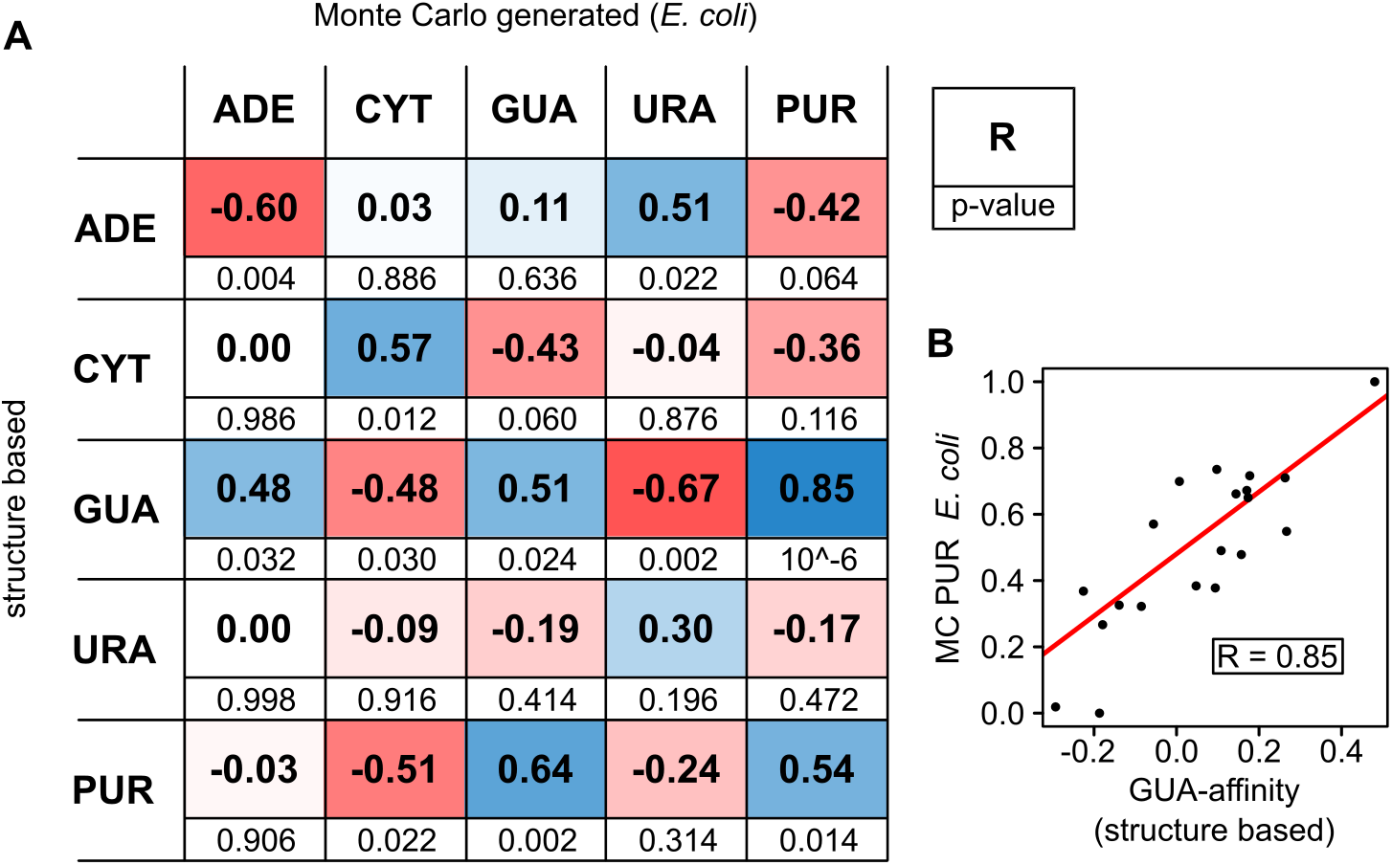
Comparison between MC-optimized scales and physical affinity scales. A) Pearson Rs (bold, top) and the associated p-values (regular, bottom) between all the MC-optimized scales derived from *E*. *coli* and the published knowledge-based nucleobase/amino-acid affinity scales [6]; B) comparison between the optimal scale derived from matching the mRNA PUR-density profiles in *E*. *coli* and the knowledge-based GUA-affinity scale (Pearson R = 0.85 between the two).

Previously, several complete interaction scales for nucleobase/amino-acid interactions have been reported from different groups [23–26]. While some of these scales focus on the general affinity of amino acids for RNAs, but do not differentiate between specific nucleobases, other scales report on the specific propensities of the 20 amino acids for each of the four RNA nucleobases. The latter, for example, include scales derived in different ways including chromatographic experiments [7], absolute binding free energy calculations [9], classical and quantum-mechanical interaction enthalpy calculations [26], knowledge-based analysis of nucleobase/amino-acid contacts derived from X-ray and NMR structures [6] and simulated partitioning experiments [10,11]. Here, we focus on the knowledge-based scales as they are arguably the most relevant proxies for the nucleobase/amino-acid affinities at the realistic RNA/protein interfaces. In Fig 4A, we show the Pearson Rs between each of MC-derived scale for the *E*. *coli* dataset with each of the physical, knowledge-based scales derived by Polyansky and Zagrovic [6]. Below the correlation coefficients, we list the p-value capturing the statistical significance for the specific correlations. Importantly, three out of five pairs of the corresponding scales (GUA, CYT, PUR) exhibit Pearson Rs > 0.5 and p-values ≤ 0.024 each, suggesting high statistical significance. Moreover, the URA scales also exhibit a positive correlation coefficient (R = 0.30, p-value = 0.098), albeit not as strong as the other three pairs. Interestingly, the knowledge-based ADE-affinity scale displays a strong anti-correlation with its MC-generated counterpart. This anti-correlating behavior is also seen if one focuses on PURs only. Namely, although ADE is a purine base, its affinity scale correlates inversely with the values for MC-generated PUR scale. On the other hand, the GUA-affinity knowledge-based scale shows by far the strongest correlation with the generated PUR scales: the relative preferences of amino acids when it comes to interaction with GUA in RNA-protein structures show values very similar to those obtained by our MC procedure, which only considers the matching between mRNA PUR-density profiles and appropriately weighted cognate protein profiles. The two correlate with a Pearson R of 0.85 and no major outliers (Fig 4B).

We have also calculated the combined p-values from the two-tailed values given in Fig 4 for two different combinations of entries in the table. In the case of the Fisher method for combining p-values, the individual tests need to be independent from each other [21,22]. Arguably, the closest subset to this requirement is the combination of the four individual diagonal elements, entailing a comparison between the corresponding scales for individual bases. This set results in a highly significant combined p-value of 1.7 x 10^−4^. Moreover, when including all combinations of URA, CYT, ADE GUA and PUR scales, a significance level of 8.3 x 10^−11^ is reached. Here, it should also be noted that there exist other suboptimal scales, which correlate better with the knowledge-based scales than do the optimal scales. For example, in the case of the *E*.*coli* PUR scale vs. the knowledge-based GUA scale, the highest correlation achieved between the two is R = 0.94, which is obtained for a suboptimal scale that itself results in an average proteome-wide correlation of <R> = -0.79. A complete analysis of suboptimal scales and their relationships with knowledge-based scales is presented in S2 Table. In general, the fact that our optimization scheme produces scales that are similar to the physically realistic nucleobase/amino-acid binding affinity scales shows that our sampling is thorough and, more importantly, suggests that compositional matching of mRNA and protein sequence profiles may indeed be related to binding between them.

**Fig 5:**
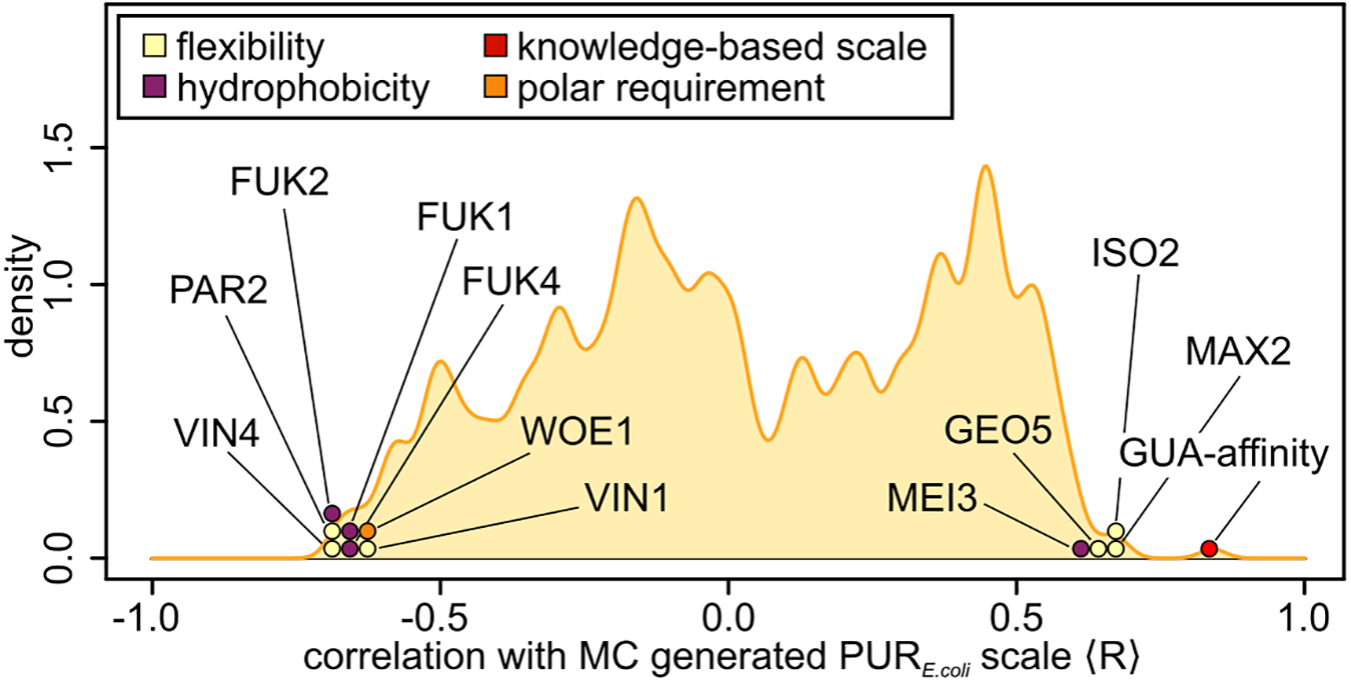
Comparison with other amino-acid properties. Distribution of Pearson Rs between all the AAindex [27,28] scales and the optimal scale derived from matching the mRNA PUR-density profiles in *E*. *coli*, with all scales showing a correlation |R|>0.60 explicitly marked: GUA-affinity scale by Polyansky et al. [6]; WOE1, a pyridine affinity scale derived by Woese et al. [29]; GEO5, a scale capturing the properties of linker regions in proteins [41]; ISO2, a flexibility scale characterizing bends in proteins [40]; MAX2, a predictor for protein backbone topology [39]; PAR2, a scale related to X-ray B-factor values of residues [38]; VIN1 & VIN4: scales assessing the correct flexibility prediction in proteins [37]; MEI3, a scale describing the effect of protein size on residue hydrophobicity [42]; FUK1, FUK2 & FUK4, scales capturing the protein surface composition of several bacterial proteins [43].

In addition, a total of 544 different one-dimensional scales [27,28], capturing different physicochemical properties of amino acids including size, hydrophobicity or interaction propensities, have been compared with the scales derived in this work by calculating the Pearson R between them. The result is visualized in Fig 5 in the case of the *E*. *coli* PUR scale. Here, it should be noted that the sign of correlation in this comparison depends only on the definition of a given scale and does not carry additional significance: for example, a hydrophilicity scale may be defined as a hydrophobicity scale and result in the same absolute result, but with an inverted sign. In general, the majority of the scales do not correlate closely with the MC-derived scales, but most of those that do are indeed in some way related to RNA/protein interactions or, interestingly, protein structural disorder. For example, the strongest correlation (R = 0.85) is obtained for the knowledge-based GUA-affinity scale [6], while the Woese pyridine affinity scale [29] ranks among the top 3% of all scales and exhibits an R = -0.63 with the MC-derived PUR-based scale. Parenthetically, a potential explanation for the observed lower density of Pearson Rs around 0 (Fig 5) may be that approximately 1/3 of all physical scales examined are related to amino-acid hydrophobicity [4]. Since our optimized PUR scale in general correlates negatively with hydrophobicity scales i.e. positively with hydrophilicity scales, this could create a somewhat lower density of Pearson Rs around 0.

## Discussion

In the present work, we have sampled the space of amino-acid scales using as a fitness function the proteome-average matching between the mRNA nucleobase density profiles and the scale-weighted sequence profiles of cognate proteins. Importantly, our framework disregards all available biological information except the sequences of the two biopolymers and approaches the amino-acid scales from an abstract perspective as arrays of 20 numerical weights that can be chosen arbitrarily. This provides the benefit that the physicochemical interpretation of the scales does not need to be given *a priori*. Rather, the features of the fitness landscape of scales provide the constraints that any physical scales need to fulfill in order to be consistent with cognate mRNA/protein sequence complementarity. At the same time, effects like codon-usage bias are included by default. Utilizing this method, we have shown that it is possible to identify scales, which are highly optimized to lead to pronounced complementarity, and that our method is highly efficient at doing so.

Notably, it was not *a priori* clear that a simple MC search would result in unique optimal solutions for the matching problem. A remarkable result that virtually one single scale is the sole result of numerous independent simulations suggests that the intrinsic features of the underlying fitness landscape guide the development of these affinity scales towards a narrow range of values. Against our expectations, especially considering the vast combinatorial space the scales reside in, evolution of highly optimized scales could be performed in less than 200 MC moves (S1 Fig). This result on its own provides some pertinent information about the space the scales reside in. Namely, such a rapid and reproducible convergence can only be explained by a landscape that is shaped like a funnel. In this picture, the continuous downhill slope guides the search reproducibly towards the final optimized scale. Although simulated annealing was included in the MC approach, this would not suffice to reach the exact same minimum in every MC run: if there existed another local minimum separated by a significant barrier, we would also have sampled it. A potential criticism of this interpretation could be that we start all our MC runs from the same scale (all weights equal to 0), which in principle could bias the final outcome. However, the MC runs initiated with different random number seeds decorrelate rapidly from each other in a few steps (as seen in the standard deviations in Fig 2B), only to converge again at the end of the runs. This, in effect, suggests that regardless of the starting point on the landscape, the MC searches reproducibly end up in the same minimum.

Our MC procedure has also resulted in a large number of suboptimal scales at all values of the average Pearson R between ~0.3 and -1. We have applied a hierarchical clustering algorithm to this data set and identified clusters of mutually similar scales. As reported in Fig 3, the development of the number of clusters as a function of the degree of matching exhibits a funnel-like shape. The important point to make is that the funnel is shallow and wide even up to the <R> values of -0.7 or higher. This means that there exist many rather different scales, which could lead to suboptimal, yet still relatively high levels of proteome-average complementary matching. This carries significant implications for our previous investigations of the complementarity hypothesis. As a case in point, our first report on the hypothesis involved a computationally derived scale of amino-acid affinity for PYR mimetics, which had led to a value of <R> of -0.74 across the human proteome [4]. This was interpreted as a strong signal of putative complementary interactions between mRNAs and their cognate proteins. On the other hand, our present analysis shows that there exist over 200 clusters of scales, whereby members of different clusters exhibit a correlation of at most 0.8 or less, all leading to a proteome-average correlation better than -0.74. In other words, the PYR-mimetic affinity scale used in our original study is by no means unique in its ability to lead to relatively high matching. While these findings do call for caution, it should be emphasized that they in no way contradict our previous interpretations. Namely, our present study only enumerates the list of possible scales that could lead to high complementarity. What is more, this list represents only a minor fraction of the space of all possible scales as indicated by our present and previous randomization studies [4,6,14]. The matching, in other words, is not a consequence of just any scale, but rather it can be achieved by only a select few, however mutually different from each other they may be.

The centerpiece of the present study is the comparison of the computationally-derived optimal scales with the published knowledge-based scales derived from structural data [6]. On the one hand, the computational scales are derived from a singular requirement that, when protein sequences are weighted by them, they yield profiles that resemble the cognate mRNA nucleobase-density profiles. Conversely, the knowledge-based scales are derived from the contact statistics of nucleobases and amino-acid side chains at the RNA-protein interfaces. They, therefore, report on the intrinsic binding preferences of the two sets of monomers. It is remarkable that Pearson correlation coefficients of up to 0.85 with highly significant p-values can be achieved between the two (Fig 4). The same can be said for the high overall p-values resulting from combining multiple scales. In other words, we start here from a simple computational exercise in which we search for scales that when applied to protein sequences yield profiles that match their cognate mRNA’s nucleobase density profiles. The fact that the scales obtained in this way resemble the nucleobase/amino-acid binding affinity scales strongly suggests that profile matching and binding indeed may be related, as put forth by the complementarity hypothesis. However, the rather weak correlation with some other affinity scales shall not be ignored. A question remains as to why protein affinity profiles for GUA strongly match the mRNA PUR-density profiles, but not as well the mRNA GUA-density profiles. In line with this, why are the protein ADE-affinity profiles inversely related to the mRNA PUR-density profiles? It has been suggested that the ADE and URA nucleobases may be newer additions to biological systems, while the GUA and CYT may have been the very first nucleobases adopted [5]. If both the matching behavior as postulated by the stereochemical hypothesis and the assumed timeline of RNA evolution hold true, this would suggest that the usage of ADE as an anti-matching base could have served a biologically important purpose. Namely, the presence of ADE has the potential to negatively regulate the level of complementarity and, therefore, the strength of binding between cognate partners, as previously suggested [5].

The present work accounts only for sequence data on both the mRNA and the protein side, with no secondary or higher-order structure elements being considered. This, of course, does not rule out structured mRNAs and proteins as interaction partners, but certainly limits the generality of the current work. It should, however, be noted that the binding between unstructured RNAs and intrinsically disordered proteins belongs to an important, large class of RNA-protein interactions and, moreover, provides a relevant context in which to look for cognate interactions [30–36]. In this regard, it may be potentially informative that some of the closest physically realistic scales to the optimal MC scales derived herein are linked with protein disorder (Fig 5) [37–43]. Finally, our results suggest that the degree of cognate mRNA-protein complementarity is heavily determined by the intrinsic binding affinities of just a handful of nucleobase/amino-acid pairs. For example, the opposite behavior of Phe and Glu/Lys define a large fraction of the PUR-density/PUR-affinity matching. At the same time, the nucleobase-binding preferences of other amino acids are much more ambiguous and diverse, even at relatively high levels of matching. The main biological implication of this is that it defines constraints on the mechanism by which genetic encoding could have evolved from binding, as proposed by the stereochemical hypothesis and our generalization of it. Specifically, the establishment of a coding relationship between codons and amino acids, which would be a consequence of complementary binding between cognate mRNA/protein pairs, is possible only for a narrow set of nucleobase-affinity values for several key residues, as defined by our study. Future research should provide information about this and other related open questions.

## Supporting information

S1 Fig Development of <R> as a function of the number of MC steps in the case of PUR-based scales. (TIF)

S2 Fig Scale value distributions for all scales and organisms investigated. Distributions of scale values for the most optimized scale of each individual MC simulation. The ordering of amino-acid symbols corresponds to the ordering of the means of the respective distributions. Results for ADE, CYT, GUA, URA and PUR are shown for each of the three organisms investigated–*E*. *coli*, *M*. *jannaschii* and *S*. *cervisiae*. The average level of matching over all scales contributing to a distribution is given in the plot.
(PDF)

S1 Table Comparison between the MC-optimized scales and the codon-based scales. Simple scales obtained from the average nucleobase-content of the codons of individual amino acids as weighted by their codon usage bias. Pearson correlation coefficients R between the scales are given on the right. Correlations of scales obtained for different organisms are reported at the end.
(PDF)

S2 Table Comparison of suboptimal scales which best match the knowledge-based scales. For each type of scale, a specific MC scale was selected that resembles the corresponding knowledge-based scale most closely. For these scales, the Pearson correlation coefficients R with the knowledge-based scales and the average, proteome-wide correlation coefficients that result by their application, are reported.
(PDF)

S1 Dataset Datasets employed in this study. Each of the original whole proteome mRNA/protein sequence datasets of *E*. *coli*, *M*. *jannaschii* and *S*. *cervisiae* used in this study in a space-separated text format.
(ZIP)
