# Supplementary figures and images for: mRNA/protein sequence complementarity and its determinants: The impact of affinity scales
Source: PLoS Comput Biol. 2017 Jul 27;13(7):e1005648. doi: 10.1371/journal.pcbi.1005648 (PMC5549747; doi:10.1371/journal.pcbi.1005648)

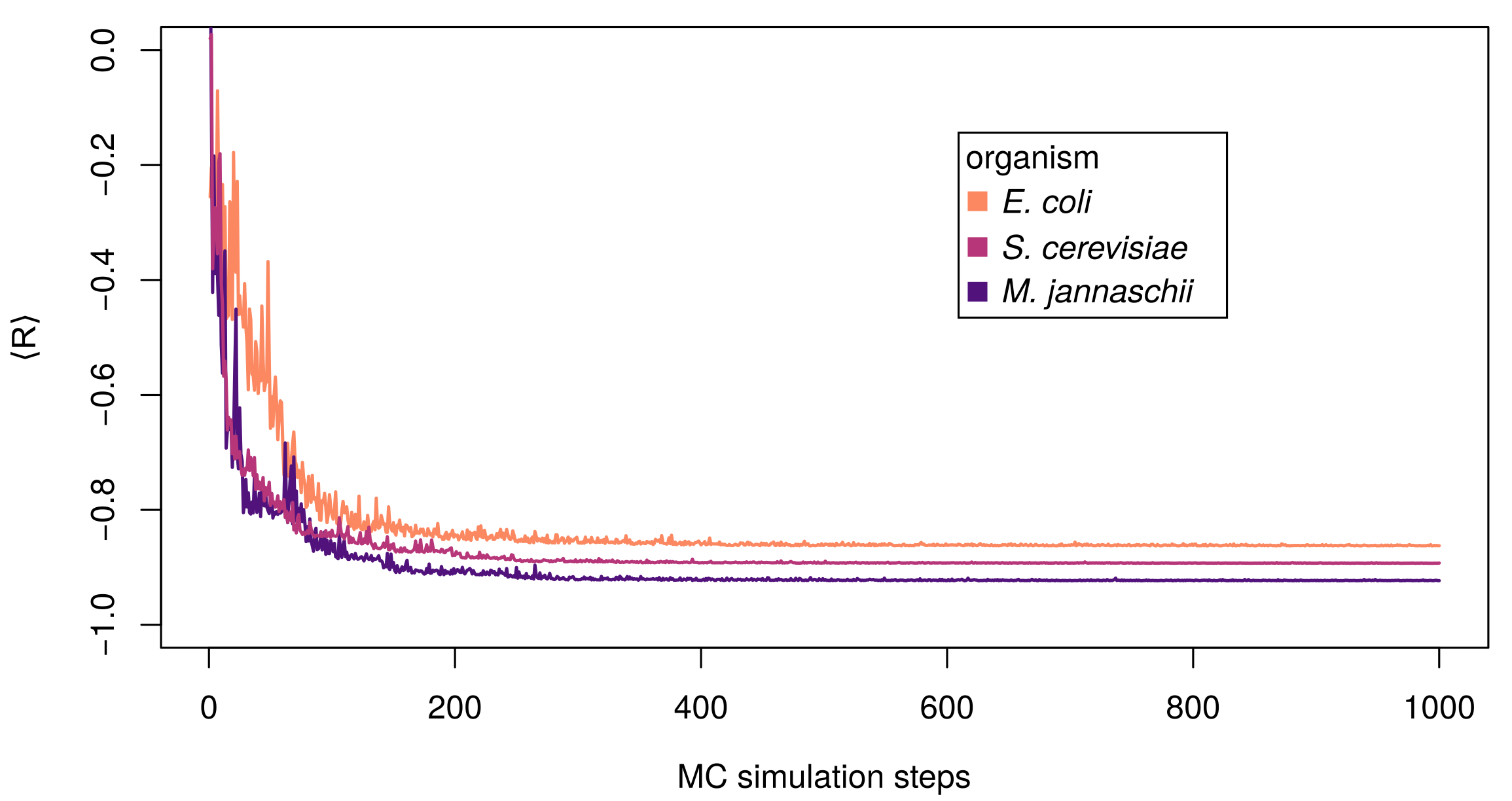

Supplement: S1 Fig — (TIF) [file pcbi.1005648.s001.tif]

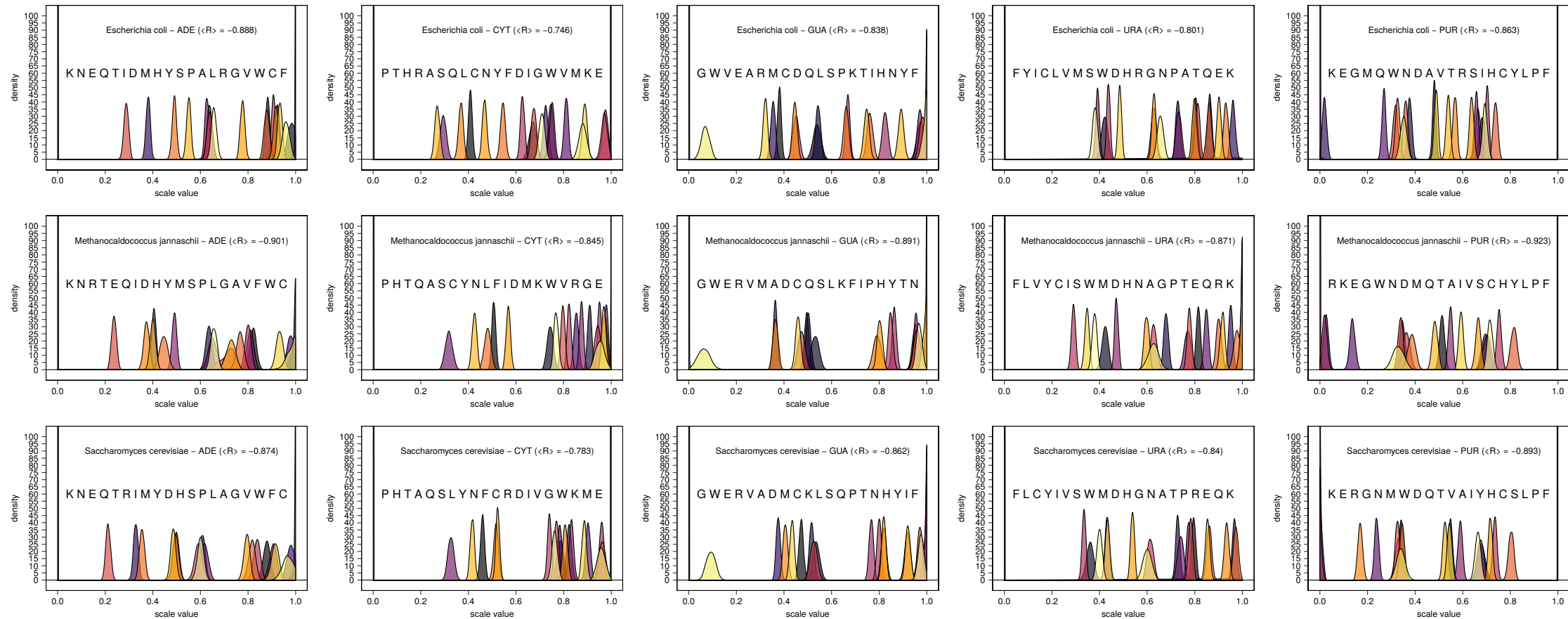

Supplement: S2 Fig — Distributions of scale values for the most optimized scale of each individual MC simulation. The ordering of amino-acid symbols corresponds to the ordering of the means of the respective distributions. Results for ADE, CYT, GUA, URA and PUR are shown for each of the three organisms investigated–E. coli, M. jannaschii and S. cervisiae. The average level of matching over all scales contributing to a distribution is given in the plot. (PDF) [file pcbi.1005648.s002.pdf]
