# Supplementary material for: mRNA/protein sequence complementarity and its determinants: The impact of affinity scales
Source: PLoS Comput Biol. 2017 Jul 27;13(7):e1005648. doi: 10.1371/journal.pcbi.1005648 (PMC5549747; doi:10.1371/journal.pcbi.1005648)
Supplement: S1 Table — Simple scales obtained from the average nucleobase-content of the codons of individual amino acids as weighted by their codon usage bias. Pearson correlation coefficients R between the scales are given on the right. Correlations of scales obtained for different organisms are reported at the end. (PDF) [file pcbi.1005648.s003.pdf]

E.coli

	Scale	A	C	D	E	F	G	H	I	K	L	M	N	P	Q	R	S	T	V	W	Y	correlation (R)
Monte Carlo	ADE	0.886	0.988	0.640	0.385	1.000	0.935	0.649	0.642	0.000	0.916	0.644	0.298	0.885	0.503	0.922	0.782	0.560	0.937	0.958	0.663	0.998
Simple		0.924	1.000	0.639	0.389	1.000	0.962	0.639	0.615	0.000	0.941	0.639	0.278	0.931	0.513	0.946	0.802	0.593	0.945	1.000	0.639	
Monte Carlo	CYT	0.406	0.665	0.749	1.000	0.720	0.821	0.295	0.749	0.981	0.622	0.973	0.671	0.000	0.543	0.361	0.464	0.262	0.883	0.880	0.699	0.989
Simple		0.401	0.736	0.824	1.000	0.799	0.808	0.326	0.801	1.000	0.600	1.000	0.739	0.000	0.529	0.363	0.528	0.321	0.898	1.000	0.796	
Monte Carlo	GUA	0.385	0.542	0.557	0.361	0.996	0.000	0.990	0.982	0.834	0.668	0.454	0.991	0.769	0.675	0.447	0.750	0.894	0.317	0.062	1.000	0.996
Simple		0.368	0.535	0.535	0.391	1.000	0.000	1.000	1.000	0.892	0.707	0.535	1.000	0.753	0.696	0.482	0.728	0.875	0.361	0.070	1.000	
Monte Carlo	URA	0.866	0.429	0.736	0.964	0.000	0.810	0.735	0.398	1.000	0.437	0.639	0.823	0.872	0.937	0.804	0.632	0.903	0.494	0.661	0.380	0.994
Simple		0.938	0.440	0.757	1.000	0.000	0.869	0.779	0.413	1.000	0.472	0.611	0.827	0.939	1.000	0.850	0.664	0.936	0.511	0.611	0.391	
Monte Carlo	PUR	0.481	0.681	0.379	0.019	1.000	0.272	0.665	0.668	0.000	0.698	0.315	0.355	0.736	0.331	0.566	0.631	0.534	0.490	0.363	0.697	0.997
Simple		0.477	0.667	0.333	0.000	1.000	0.248	0.667	0.644	0.000	0.736	0.333	0.333	0.759	0.333	0.579	0.622	0.535	0.491	0.333	0.667	

M.janaschii

	Scale	A	C	D	E	F	G	H	I	K	L	M	N	P	Q	R	S	T	V	W	Y	correlation (R)
Monte Carlo	ADE	0.825	0.983	0.635	0.410	0.987	0.822	0.678	0.495	0.000	0.809	0.730	0.238	0.764	0.451	0.366	0.730	0.411	0.933	1.000	0.656	0.992
Simple		0.848	1.000	0.630	0.407	1.000	0.792	0.630	0.470	0.000	0.762	0.630	0.260	0.754	0.394	0.370	0.748	0.449	0.919	1.000	0.630	
Monte Carlo	CYT	0.495	0.732	0.904	1.000	0.852	0.977	0.317	0.870	0.947	0.818	0.940	0.791	0.000	0.471	0.977	0.559	0.422	0.966	0.949	0.765	0.990
Simple		0.461	0.843	0.914	1.000	0.898	0.967	0.371	0.950	1.000	0.861	1.000	0.856	0.000	0.510	0.988	0.598	0.458	0.967	1.000	0.888	
Monte Carlo	GUA	0.506	0.539	0.502	0.373	0.961	0.000	0.973	0.955	0.862	0.844	0.487	1.000	0.954	0.801	0.365	0.813	0.993	0.465	0.047	0.975	0.998
Simple		0.518	0.538	0.538	0.354	1.000	0.000	1.000	1.000	0.863	0.897	0.538	1.000	0.981	0.833	0.416	0.825	0.980	0.499	0.075	1.000	
Monte Carlo	URA	0.822	0.435	0.690	0.961	0.000	0.851	0.767	0.475	0.995	0.294	0.628	0.786	0.905	0.978	1.000	0.610	0.931	0.351	0.642	0.382	0.993
Simple		0.841	0.399	0.705	1.000	0.000	0.926	0.743	0.476	1.000	0.337	0.642	0.747	0.909	1.000	0.997	0.599	0.870	0.417	0.642	0.366	
Monte Carlo	PUR	0.516	0.682	0.340	0.020	1.000	0.124	0.695	0.549	0.013	0.754	0.353	0.336	0.809	0.371	0.000	0.661	0.479	0.591	0.333	0.711	0.998
Simple		0.515	0.667	0.333	0.000	1.000	0.091	0.667	0.522	0.000	0.711	0.333	0.333	0.765	0.333	0.011	0.646	0.489	0.565	0.333	0.667	

S.cerevisiae

	Scale	A	C	D	E	F	G	H	I	K	L	M	N	P	Q	R	S	T	V	W	Y	correlation (R)
Monte Carlo	ADE	0.890	0.998	0.617	0.337	1.000	0.918	0.616	0.506	0.000	0.846	0.607	0.218	0.825	0.366	0.507	0.805	0.505	0.928	0.978	0.615	0.999
Simple		0.885	1.000	0.613	0.342	1.000	0.913	0.613	0.505	0.000	0.837	0.613	0.226	0.843	0.348	0.523	0.812	0.494	0.915	1.000	0.613	
Monte Carlo	CYT	0.462	0.791	0.834	1.000	0.781	0.912	0.328	0.834	0.965	0.744	0.961	0.775	0.000	0.522	0.811	0.525	0.416	0.887	0.969	0.767	0.998
Simple		0.434	0.826	0.839	1.000	0.812	0.909	0.371	0.880	1.000	0.768	1.000	0.814	0.000	0.537	0.828	0.540	0.439	0.907	1.000	0.800	
Monte Carlo	GUA	0.475	0.540	0.516	0.372	1.000	0.000	0.973	1.000	0.766	0.803	0.532	0.973	0.931	0.821	0.396	0.826	0.923	0.433	0.095	0.979	0.999
Simple		0.476	0.529	0.529	0.388	1.000	0.000	1.000	1.000	0.804	0.816	0.529	1.000	0.942	0.852	0.409	0.825	0.935	0.438	0.058	1.000	
Monte Carlo	URA	0.796	0.364	0.730	0.961	0.000	0.766	0.727	0.426	1.000	0.340	0.620	0.785	0.855	0.973	0.939	0.540	0.856	0.436	0.584	0.402	0.997
Simple		0.858	0.374	0.749	1.000	0.000	0.824	0.752	0.437	1.000	0.350	0.615	0.769	0.880	1.000	0.946	0.557	0.868	0.466	0.615	0.396	
Monte Carlo	PUR	0.548	0.668	0.342	0.005	1.000	0.234	0.672	0.583	0.000	0.734	0.325	0.325	0.798	0.338	0.171	0.714	0.525	0.537	0.337	0.664	0.999
Simple		0.530	0.667	0.333	0.000	1.000	0.218	0.667	0.573	0.000	0.729	0.333	0.333	0.823	0.333	0.170	0.714	0.518	0.529	0.333	0.667	

Correlation between organisms

	ADE	CYT	GUA	URA	PUR
E.coli vs *M.janaschii*	0.878	0.864	0.968	0.968	0.869
E.coli vs *S.cerevisiae*	0.940	0.927	0.976	0.979	0.930
M.janaschii vs *S.cerevisiae*	0.971	0.981	0.993	0.987	0.982
