# Supplementary material for: mRNA/protein sequence complementarity and its determinants: The impact of affinity scales
Source: PLoS Comput Biol. 2017 Jul 27;13(7):e1005648. doi: 10.1371/journal.pcbi.1005648 (PMC5549747; doi:10.1371/journal.pcbi.1005648)
Supplement: S2 Table — For each type of scale, a specific MC scale was selected that resembles the corresponding knowledge-based scale most closely. For these scales, the Pearson correlation coefficients R with the knowledge-based scales and the average, proteome-wide correlation coefficients that result by their application, are reported. (PDF) [file pcbi.1005648.s004.pdf]

E. coli

compared scales	Pearson R	scale fitness
ADE_{KB} vs ADE_{MC}	0.719	-0.048
CYT_{KB} vs CYT_{MC}	0.868	-0.556
GUA_{KB} vs GUA_{MC}	0.803	-0.540
URA_{KB} vs URA_{MC}	0.818	-0.491
PUR_{KB} vs PUR_{MC}	0.862	-0.736
GUA_{KB} vs PUR_{MC}	0.943	-0.789

M. jannaschii

compared scales	Pearson R	scale fitness
ADE_{KB} vs ADE_{MC}	0.699	0.314
CYT_{KB} vs CYT_{MC}	0.912	-0.472
GUA_{KB} vs GUA_{MC}	0.823	-0.322
URA_{KB} vs URA_{MC}	0.787	-0.517
PUR_{KB} vs PUR_{MC}	0.864	-0.767
GUA_{KB} vs PUR_{MC}	0.918	-0.865

S. cerevisiae

compared scales	Pearson R	scale fitness
ADE_{KB} vs ADE_{MC}	0.676	-0.200
CYT_{KB} vs CYT_{MC}	0.866	-0.542
GUA_{KB} vs GUA_{MC}	0.772	-0.347
URA_{KB} vs URA_{MC}	0.807	-0.346
PUR_{KB} vs PUR_{MC}	0.839	-0.729
GUA_{KB} vs PUR_{MC}	0.947	-0.843

KB … knowledge based (refers to scales published in Polyansky AA, Zagrovic B. Evidence of direct complementary interactions between messenger RNAs and their cognate proteins. Nucleic Acids Res. 2013;41: 8434–8443. as Set 2+)

MC … Monte Carlo generated – closest matching scales found in the total set of scales generated

scale fitness … mean whole proteome correlation resulting from the given scales applied on the respective proteome. Negative values show better interaction according to the definition of affinities. For calculation details please refer to the Materials and Methods section.
